# The Pollutant Diethylhexyl Phthalate Regulates Hepatic Energy Metabolism via Species-Specific PPARα-Dependent Mechanisms

**DOI:** 10.1289/ehp.0901217

**Published:** 2009-10-08

**Authors:** Jérôme N. Feige, Alan Gerber, Cristina Casals-Casas, Qian Yang, Carine Winkler, Elodie Bedu, Manuel Bueno, Laurent Gelman, Johan Auwerx, Frank J. Gonzalez, Béatrice Desvergne

**Affiliations:** 1 Center for Integrative Genomics, National Research Center “Frontiers in Genetics,” University of Lausanne, Lausanne, Switzerland; 2 Laboratory of Metabolism, Center for Cancer Research, National Cancer Institute, National Institutes of Health, Department of Health and Human Services, Bethesda, Maryland, USA; 3 Ecole Polytechnique Fédérale de Lausanne, Lausanne, Switzerland

**Keywords:** DEHP, endocrine disruptor, metabolism, PPAR, species specificity

## Abstract

**Background:**

The modulation of energetic homeostasis by pollutants has recently emerged as a potential contributor to the onset of metabolic disorders. Diethylhexyl phthalate (DEHP) is a widely used industrial plasticizer to which humans are widely exposed. Phthalates can activate the three peroxisome proliferator–activated receptor (PPAR) isotypes on cellular models and induce peroxisome proliferation in rodents.

**Objectives:**

In this study, we aimed to evaluate the systemic and metabolic consequences of DEHP exposure that have remained so far unexplored and to characterize the underlying molecular mechanisms of action.

**Methods:**

As a proof of concept and mechanism, genetically engineered mouse models of PPARs were exposed to high doses of DEHP, followed by metabolic and molecular analyses.

**Results:**

DEHP-treated mice were protected from diet-induced obesity via PPARα-dependent activation of hepatic fatty acid catabolism, whereas the activity of neither PPARβ nor PPARγ was affected. However, the lean phenotype observed in response to DEHP in wild-type mice was surprisingly abolished in PPARα-humanized mice. These species differences are associated with a different pattern of coregulator recruitment.

**Conclusion:**

These results demonstrate that DEHP exerts species-specific metabolic actions that rely to a large extent on PPARα signaling and highlight the metabolic importance of the species-specific activation of PPARα by xenobiotic compounds.

The prevalence of obesity and its associated metabolic complications has dramatically increased during the past decades; it has been suggested that this could, to some extent, be linked to the exposure to environmental pollutants, which coincidently increased during the same period (Baillie-Hamilton 2002; Grun and Blumberg 2007; Heindel 2003; Newbold et al. 2007). Endocrine disruptors constitute a wide class of chemicals that can affect human and animal populations by interfering with the synthesis, elimination, and mechanisms of action of hormones (Markey et al. 2002; Waring and Harris 2005). One of their key mechanisms of action is the modulation of gene expression programs by targeting the activity of a family of nuclear receptors that are activated by intracellular lipophilic hormones and mediators. The concept of endocrine disruption, which initially arose because of the interference with reproductive biology, is now gradually being broadened to other receptors implicated in different aspects of homeostasis (Tabb and Blumberg 2006).

Metabolic homeostasis requires a controlled balance between energy storage and use, and several nuclear receptors as well as their coregulators are instrumental in regulating these processes (Desvergne et al. 2006; Feige and Auwerx 2007). Among these, peroxisome proliferator–activated receptors (PPARs) play a prominent role by acting as lipid sensors that cooperate in different organs to adapt gene expression to a given metabolic status (Desvergne et al. 2004; Evans et al. 2004; Feige et al. 2006). PPARα (NR1C1) is the founding member of the family and was initially isolated as the receptor inducing the hepatic proliferation of peroxisomes in rodents in response to synthetic chemicals. However, this function represents only a small subset of the physiologic functions regulated by this receptor. PPARα and PPARβ/δ (NR1C2, referred to here as PPARβ) share partially overlapping functions in the control of catabolic metabolism by promoting fatty acid oxidation in tissues with high metabolic rates such as liver or muscle (Desvergne et al. 2004; Evans et al. 2004; Feige et al. 2006). In contrast, PPARγ (NR1C3) controls fat storage in adipose tissue by promoting differentiation and survival of adipocytes and also plays major roles in the control of insulin sensitivity (Lehrke and Lazar 2005).

Several endocrine disruptors, including pesticides, industrial solvents, and plasticizers, can activate PPARs in cellular models (Bility et al. 2004; Grun et al. 2006; Hurst and Waxman 2003; Kanayama et al. 2005; Lapinskas et al. 2005; Takeuchi et al. 2006). However, the metabolic consequences of PPAR activation by endocrine disruptors remain largely unknown, although some physiologic consequences are emerging, such as adipogenic action of organotins (Grun et al. 2006) and PPARα-dependent induction of hepatic peroxisome proliferation by phthalates (Lapinskas et al. 2005; Ward et al. 1998). Diethylhexyl phthalate (DEHP) is among the phthalate esters most abundantly used as industrial plasticizers and is also found in cosmetics, as well as in industrial paints and solvents. DEHP is found in flexible plastics used for manufacturing a wide variety of daily products, including medical devices and food packaging, and its propensity to leach can lead to high levels of human exposure [National Toxicology Program Center for the Evaluation of Risks to Human Reproduction (NTP-CERHR) 2000]. When ingested through contaminated food, DEHP is converted by intestinal lipases to its monoester equivalent monoethylhexyl phthalate (MEHP), which is then preferentially absorbed (Huber et al. 1996). MEHP can then be metabolized into a myriad of secondary metabolites that can be excreted (Rusyn et al. 2006), among which 2-ethylhexanoic acid has also been reported as a low-affinity PPARα activator (Lapinskas et al. 2005; Maloney and Waxman 1999). The biological effects of exposure to DEHP are hence of major concern, but their possible adverse effects on human health remain obscure.

These observations led us to investigate the molecular aspects of phthalate-mediated activation of PPARγ. In cellular models, MEHP induces adipogenesis by modulating PPARγ activity (Feige et al. 2007), suggesting that DEHP could promote obesity *in vivo* if its MEHP metabolite reaches adipose tissue (Grun and Blumberg 2007; Heindel 2003). However, MEHP also activates PPARα and PPARβ *in vitro* (Bility et al. 2004; Feige et al. 2007; Hurst and Waxman 2003; Lapinskas et al. 2005), suggesting that DEHP exposure may also promote fatty acid oxidation. In the present study, we addressed the metabolic consequences of DEHP exposure in mice. Intriguingly, DEHP exposure protected mice from weight gain under both a regular diet and a diet containing high fat content; this effect could be attributed solely to the hepatic oxidative functions of PPARα. The lean phenotype of DEHP-treated mice was not observed in mice humanized for PPARα where the mouse receptor has been replaced by a human PPARα (hPPARα) transgene. At the molecular level, the functional differences between mouse PPARα (mPPARα) and hPPARα are associated with a different pattern of coregulator recruitment in the presence of MEHP. Altogether, our observations highlight key physiopathologic consequences of chronic exposure to DEHP and highlight the metabolic importance of the species-specific metabolic consequences of PPARα activation by xenobiotics compounds.

## Materials and Methods

### Animal experiments

We carried out DEHP exposure in wild-type (WT) mice using C57Bl6J mice (Charles River, L’Arbresle, France), which were fed a chow diet (CD) or a high-fat diet (HFD) containing 60% fat (Provimi-Kliba, Kaiseraugst, Switzerland), supplemented with 10 mL sunflower oil (vehicle) per kilogram of feed in the presence or absence of DEHP (Sigma Aldrich, Buchs, Switzerland). Genetically engineered mouse models are described in the Supplemental Material available online (doi:10.1289/ehp.0901217.S1 via http://dx.doi.org/). Animal experiments were conducted humanely and with regard for alleviation of suffering, and were approved by the relevant animal welfare commissions.

### Metabolic phenotyping

We performed glucose tolerance tests after an intraperitoneal injection of 2 g glucose/kg body mass after 4 hr of fasting. Fat and lean body compositions were measured by nuclear magnetic resonance (NMR) on an Echo-MRI (magnetic resonance imaging) 100 apparatus (Echo Medical Systems, Houston, TX, USA). Indirect calorimetry in metabolic cages was performed with a Comprehensive Lab Animal Monitoring System (Columbus Instruments, Columbus, OH, USA) during 24 hr after a 36- to 48-hr acclimation period. The exercise test was performed on a treadmill (Columbus Instruments); a 6-min training period at low velocity was given 24 hr before the exercise (Feige et al. 2008; Lagouge et al. 2006). Mice were exercised until they reached fatigue [defined by resting for > 50% of the time on the shock pad; see Supplemental Material, Figure 2 (doi:10.1289/ehp.0901217.S1)]. Biochemical assays and quantitative PCR (polymerase chain reaction) techniques are described in the Supplemental Material (doi:10.1289/ehp.0901217.S1).

### Reporter gene and pull-down assays

We performed PPAR luciferase-based reporter gene assays in C2C12 mouse myoblast cells using plasmids, compounds, and procedures described previously (Feige et al. 2005, 2007). Pull-downs using glutathione *S*-transferase (GST)-labeled coregulators and ^35^S-labeled PPARα produced in reticulocyte lysates were performed as described previously (Feige et al. 2005, 2007) in the presence of vehicle, 100 μM Wy14643 (Cayman Chemicals, Ann Harbor, MI, USA), or 1,000 μM MEHP (Imperial Chemical Industries, Slough, UK). The primary culture of hepatocytes and adenoviral infections are described in the Supplemental Materials (doi:10.1289/ehp.0901217.S1).

### Statistical analyses

All data are reported as mean ± SE. Results were considered statistically significant when *p*-values were < 0.05 by unpaired two-tailed Student’s *t*-test.

## Results

### DEHP exposure protects from obesity in mice

**Figure 1:**
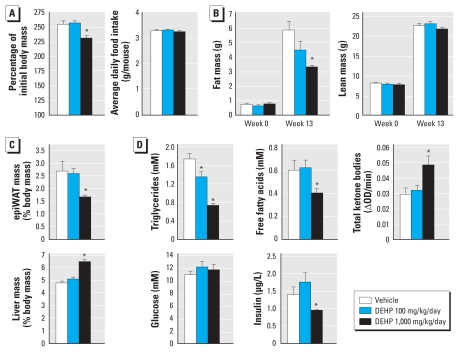
DEHP exposure induces a lean phenotype in WT mice on CD. Four-week-old WT C57Bl6J male mice were fed a CD supplemented with vehicle (10 mL/kg feed) alone or combined with DEHP for an exposure of either 100 or 1,000 mg/kg/day (*n* = 10/group). (*A*) Body mass after 13 weeks of treatment and average weekly food intake during the treatment period. (*B*) Body composition measured by MRI at the beginning and at the end of the treatment. (*C*) epiWAT and liver mass after 13 weeks of treatment. (*D*) Plasma profile after 13 weeks of treatment. OD, optical density. *p ≤ 0.05 compared with vehicle treatment.

The ability of the DEHP metabolite MEHP to activate PPARα and PPARβ, on the one hand, and PPARγ on the other, suggests that this endocrine disruptor could potentially influence the two arms of the energetic balance by affecting both energy expenditure and storage. To understand the global metabolic output of an exposure to DEHP, we fed WT C57Bl6J mice with a regular CD containing vehicle or DEHP. Animals were thereby exposed to the compound through intestinal absorption, a route that mimics one of the most frequent sources of exposure in humans and favors the conversion of DEHP to its major bioactive metabolite MEHP. DEHP was incorporated in the diet at two concentrations, leading to average exposures of 100 and 1,000 mg/kg body mass/day), dosages that were previously reported to be less than and greater than the minimal exposure required for hepatic peroxisome proliferation, respectively (David et al. 1999). The treatment started just after weaning at 3 weeks of age. At week 13, mice treated with 1,000 mg/kg/day DEHP gained approximately 15% less weight than did controls or mice treated with 100 mg/kg/day; this difference did not result from different feeding behaviors because all three groups consumed the same amount of food (Figure 1A). Consistently, the lean mass measured by NMR was not affected by the treatment at either dose, but the weight difference at the high dose was solely caused by reduced total body fat (Figure 1B). Along the same line, the mass of the epididymal white adipose tissue (epiWAT) at sacrifice was also reduced by 40% (Figure 1C). Only the high dose of DEHP induced a hepatomegaly (Figure 1C), testifying that significant peroxisome and hepatocyte proliferation occurred only at 1,000 mg/kg/day (Rusyn et al. 2006). In the blood, triglyceride and free fatty acid levels were reduced in DEHP-treated mice (Figure 1D), suggesting that the reduced fat mass of these animals reflects enhanced fatty acid oxidation. DEHP treatment consistently increased plasma total ketone bodies, a marker of hepatic fatty acid oxidation. Plasma glucose levels were not affected by DEHP in fed or fasted animals (Figure 1D and data not shown, respectively). Although DEHP treatment did not modify glucose tolerance [see Supplemental Material, Figure 1 (doi:10.1289/ehp.0901217.S1)], insulin levels were significantly lower in mice treated with 1,000 mg/kg/day DEHP (Figure 1D). Altogether, these results demonstrate that DEHP exposure reduces adiposity under CD.

**Figure 2:**
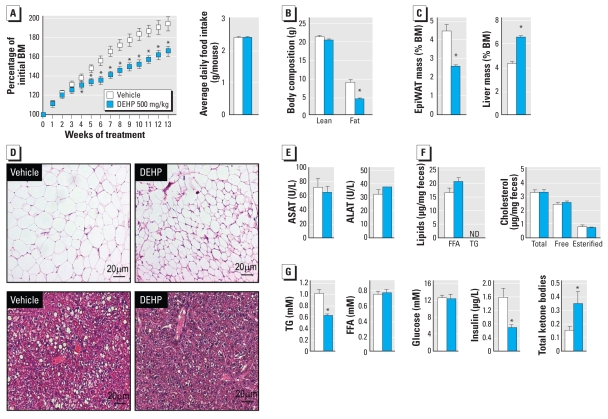
DEHP exposure induces a lean phenotype in WT mice on an HFD. Seven-week-old WT C57Bl6J male mice were fed HFD supplemented with vehicle (10 mL/kg feed) alone or combined with DEHP for an exposure of 500 mg/kg/day (*n* = 9/group). Abbreviations: ALAT, alanine aminotransferase; ASAT, aspartate aminotransferase; FFA, free fatty acids; ND, not detected; TG, triglycerides. (*A*) Growth curve and average weekly food intake during the entire treatment. (*B*) Body composition measured by MRI after 13 weeks of treatment. (*C*) epiWAT and liver mass at the end of the treatment period. (*D*) epiWAT (top) and liver (bottom) histology at the end of the treatment. (*E*) Plasma levels of ASAT and ALAT at the end of the treatment period. (*F*) Lipid and cholesterol levels in feces. (*G*) Plasma profiles of TG, FFA, glucose, insulin, and ketone bodies at the end of the treatment period. *p ≤ 0.05 compared with vehicle treatment.

To understand whether a diet high in fat would exacerbate or counterbalance this lean phenotype, we fed adult WT mice the HFD (containing 60% calories as fat) supplemented with vehicle or 500 mg/kg/day DEHP (Figure 2). The HFD alone led to a doubling of body mass (Figure 2A), essentially due to the increased fat mass from 8–10% of body mass before treatment (as measured by NMR), to 30% after 13 weeks of HFD, whereas fat mass remained at 8–10% after 13 weeks of CD (data not shown). DEHP strongly protected mice from HFD-induced obesity without affecting food intake (Figure 2A). Although hepatomegaly was observed in DEHP-treated mice because of PPARα-dependent peroxisome and hepatocyte proliferation (Rusyn et al. 2006), *in vivo* analyses of body composition revealed that the reduced body mass resulted from lower fat mass (Figure 2B,C). Consistent with its protective effect on adiposity, DEHP inhibited adipocyte hypertrophy and hepatic lipid droplet accumulation (Figure 2D). Despite peroxisome proliferation in hepatocytes in response to DEHP, the global histology of the liver was normal, with no signs of inflammation or necrosis (Figure 2D). The normal plasma levels of alanine aminotransferase and aspartate aminotransferase (Figure 2E), two markers of liver damage, further suggest that the lean phenotype of DEHP-treated mice is not the consequence of hepatic toxicity. In addition, the reduced adiposity did not result from a difference in lipid absorption or excretion, because lipid concentrations in the feces were not affected by the treatment (Figure 2F). DEHP exposure did not affect free fatty acid levels but significantly reduced triglyceride and increased ketone body concentrations, thereby suggesting a catabolic state primarily occurring in the liver (Figure 2G). As observed under the CD, insulin levels were also reduced in the blood of DEHP-treated animals, whereas glycemia was not affected.

### Metabolic phenotyping of DEHP-treated mice

**Figure 3:**
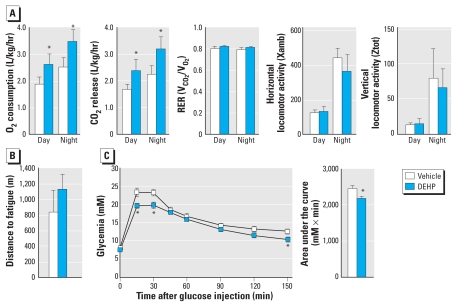
Metabolic phenotyping of DEHP-treated mice. Phenotyping tests were performed on animals fed HFD treated with vehicle (10 mL/kg feed) alone or combined with DEHP for an exposure of 500 mg/kg/day (*n* = 8 mice per group). Abbreviations: RER, respiratory exchange ratio; V, volume; Xamb, ambulatory; Ztot, rearing. (*A*) Respiratory and locomotor parameters of vehicle- and DEHP-treated mice determined during 24 hr in metabolic cages (*n* = 8 mice/group). (*B*) Endurance exercise tests performed on eight mice per group (see “Materials and Methods” for details). (*C*) Intraperitoneal glucose tolerance test with 2 g glucose/kg body mass after 4 hr of fasting. *p ≤ 0.05 compared with vehicle treatment.

Indirect calorimetry revealed that DEHP treatment increases oxygen consumption and carbon dioxide release both during day and night (Figure 3A), thus providing evidence of an increased metabolic rate in DEHP-treated mice. However, the fuel preference was not modified by the exposure to DEHP because the respiratory exchange ratio [ratio between carbon dioxide (CO_2_) release and oxygen (O_2_) consumption] was not affected. Although the protective effect of DEHP on body weight gain was not a consequence of increased spontaneous locomotor activity (Figure 3A), we evaluated muscle functions by an exercise test that evaluates the global oxidative capacities of skeletal muscle. The distance ran until fatigue did not differ between DEHP-treated mice and their controls (Figure 3B), indicating that muscle is not the primary site of MEHP action.

The active DEHP metabolite MEHP can activate the three PPAR isotypes, of which PPARγ plays a crucial role in controlling insulin sensitivity (Lehrke and Lazar 2005). A full PPARγ activation seems unlikely in DEHP-treated mice because PPARγ activation by full agonists such as thiazolidinediones promotes adipogenesis and weight gain. However, we recently reported that MEHP acts as a selective PPARγ modulator (Feige et al. 2007), a class of compounds capable of uncoupling the action of PPARγ on adipogenesis from those on insulin sensitivity and glucose tolerance (Gelman et al. 2007). We therefore measured glucose tolerance and found that DEHP administration mildly enhanced glucose tolerance (Figure 3C). However, given the detrimental action of adiposity on glucose tolerance, this effect is most likely a consequence of the lean phenotype rather than of PPARγ activation.

### DEHP activates hepatic fatty acid oxidation through PPARα

**Figure 4:**
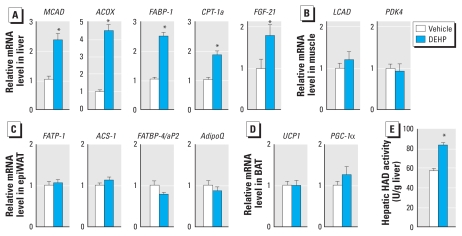
DEHP promotes hepatic fatty acid oxidation. Gene expression was determined by quantitative reverse transcriptase-PCR [see Supplemental Material (doi:10.1289/ehp.0901217.S1) for details] on RNA extracted from liver (*A*), gastrocnemius skeletal muscle (*B*), epiWAT (*C*), and brown adipose tissue (BAT) (*D*). Gene expression was normalized to three housekeeping genes (glyceraldehyde-3-phosphate dehydrogenase, β glucuronidase, and ribosomal protein S9 for the liver; eukaryotic translation elongation factor 1α1, TATA box binding protein, and ribosomal protein S9 for the other tissues). Abbreviations: *ACOX*, acyl-CoA oxidase 1; *ACS-1*, acetyl-CoA synthetase 1; *AdipoQ*, adiponectin; *CPT-1a*, carnitine palmitoyltransferase 1a (liver); *FABP-1*, fatty acid binding protein 1 (liver); *FABP-4*/*aP2*, fatty acid binding protein 4 (adipocyte); *FATP-1*, fatty acid transport protein 1; *FGF-21*, fibroblast growth factor 21; *LCAD*, long chain acyl-CoA dehydrogenase; *MCAD*, medium chain acyl-CoA dehydrogenase; *PDK4*, pyruvate dehydrogenase kinase 4; *PGC-1*α, PPARγ coactivator 1α; *UCP1*, uncoupling protein 1. (*E*) 3-Hydroxyacyl-coenzyme A dehydrogenase (HAD) activity was measured in liver extracts of DEHP-treated mice given the HFD. *p ≤ 0.05 compared with vehicle treatment.

One key question to fully understand the metabolic consequences of DEHP exposure is the individual responses of organs controlling energy expenditure and storage. Because MEHP can activate the three PPAR isotypes in cellular systems, all PPAR-expressing tissues are potential targets if they are exposed to MEHP upon DEHP ingestion. We thus measured the expression of well-characterized PPAR target genes (Figure 4). In the liver, DEHP exposure induced the expression of genes implicated in various aspects of fatty acid oxidation, including mitochondrial β-oxidation (medium chain acylCoA dehydrogenase; *MCAD*), peroxisomal β-oxidation (AcylCoA oxidase 1; *ACOX*), intracellular fatty acid shuttling (fatty acid binding protein 1; *FABP-1*), and mitochondrial fatty acid import (carnitine palmitoyl transferase 1a; *CPT-1a*) (Figure 4A). Strikingly, DEHP also robustly induced the hepatic expression of fibroblast growth factor 21 (*FGF*-*21*), a gene recently described to control lipid oxidation and ketogenesis in the liver and lipolysis in the adipose tissue (Badman et al. 2007; Inagaki et al. 2007; Kharitonenkov et al. 2005). These gene profiling experiments were functionally confirmed by the biochemical measurement of hepatic hydroxyacyl-CoA dehydrogenase activity, which demonstrated that fatty acid oxidation was increased in the liver of DEHP-treated mice (Figure 4E). Because PPARα is the most prominent hepatic PPAR isotype and all five of the genes we tested are reported to be under the direct transcriptional control of PPARα, these observations suggested that the lean phenotype of DEHP-treated mice results at least in part from hepatic fatty acid oxidation mediated by PPARα. Other energy-dissipating tissues expressing PPARs do not seem to contribute to this phenotype as oxidative target genes in skeletal muscle [long chain acyl-CoA dehydrogenase (*LCAD*) and pyruvate dehydrogenase kinase 4 (*PDK4*)] and in brown adipose tissue [uncoupling protein 1 (*UCP1*) and PPARγ coactivator 1α (*PGC-1*α)] were not affected (Figure 4B,D). In addition, the expression of adipogenic target genes under direct transcriptional control of PPARγ in white adipose tissue (WAT) was not affected (Figure 4C). These gene expression data therefore demonstrate that DEHP exposure does not affect adipogenesis *in vivo* and drives a catabolic response occurring primarily in the liver, most probably through activation of PPARα.

**Figure 5:**
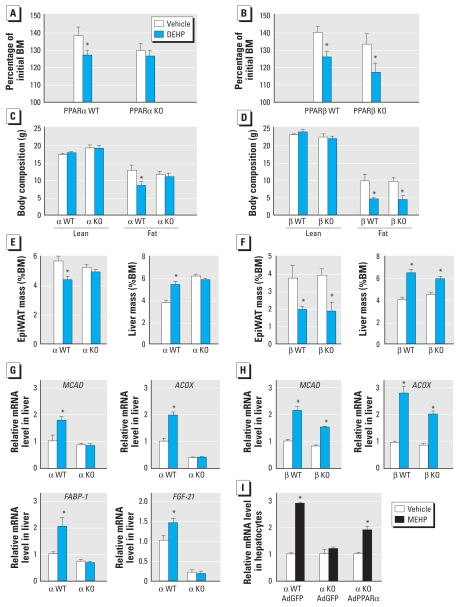
The lean phenotype of DEHP-treated mice requires PPARα but not PPARβ. PPARα (α) WT and null [knockout (KO)] mice on a pure SV129 background (*A*, *C*, *E*, *G*) and PPARβ (β) WT and KO mice on a mixed SV129/C57Bl6J background (*B*, *D*, *F*, *H*) were fed HFD and exposed to vehicle or 500 mg/kg DEHP as described in Figure 2 (*n* = 6/group). Body mass (BM; *A*, *B*) and body composition measured by MRI (*C*, *D*) were measured after 13 weeks of treatment. At the end of the treatment period, epiWAT and liver masses were analyzed (*E, F*), and liver gene expression was measured (*G, H*) as described in Figure 4. (*I*) Fold induction of *FGF-21* mRNA measured in primary hepatocytes derived from PPARα WT and KO mice, infected with adenoviruses (Ad) encoding green fluorescent protein (GFP) or mouse PPARα adenoviruses and treated with 100 μM MEHP for 12 hr. *p ≤ 0.05 compared with vehicle treatment.

Because the oxidative functions of PPARα and PPARβ partially overlap, we dissected the contribution of each individual receptor by feeding PPARα- and PPARβ-null mice the HFD supplemented with DEHP. We observed the protective effects of DEHP on weight gain in WT mice as well as in PPARβ-null mice (Figure 5A,B). In contrast, DEHP did not influence the body mass of PPARα-null mice (Figure 5A), demonstrating that the anti-obesity action of DEHP requires PPARα only. This conclusion was further corroborated by reduced fat mass after DEHP exposure in PPARβ-null mice but not PPARα-null mice (Figure 5C–F). Similarly, the DEHP-dependent hepatomegaly caused by peroxisome proliferation was PPARα- but not PPARβ-dependent (Figure 5E,F). PPARα was also indispensable to the hepatic induction of oxidative gene expression after DEHP exposure (Figure 5G), whereas the influence of PPARβ was modest to nonexistent based on the genes analyzed (Figure 5H). To further demonstrate the role of hepatic PPARα in the response to DEHP, we isolated primary hepatocytes from WT and PPARα-null mice and showed that only WT hepatocytes were responsive to MEHP. In addition, the lack of response to MEHP in PPARα-null hepatocytes was restored by adenoviral-mediated overexpression of PPARα (Figure 5I). Altogether, these results demonstrate that DEHP protects from obesity by activating the catabolic functions of PPARα in the liver.

### DEHP protects WT mice but not humanized models from obesity

**Figure 6:**
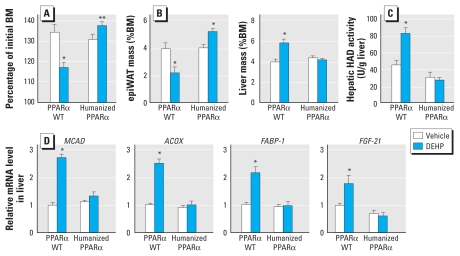
DEHP protects from obesity in mouse but not human models. PPARα WT and PPARα-humanized mice were fed HFD and exposed to vehicle or 500 mg/kg/day DEHP as described in Figure 2 (*n* = 5/group). (*A*) Body mass (BM) was measured after 13 weeks of treatment. (*B*–*D*) At the end of the treatment, epiWAT and liver masses were analyzed (*B*), and 3-hydroxyacyl-coenzyme A dehydrogenase (HAD) activity (*C*) and gene expression (*D*) were measured in the liver as described in Figure 4. *p ≤ 0.05 compared with vehicle treatment. **p = 0.08.

The identification of hepatic PPARα-dependent activation as the cause of the lean phenotype of DEHP-treated mice raises the question of how this protective action may translate to humans exposed to DEHP. In mice, peroxisome and hepatic proliferation is directly controlled by PPARα activation and coexists with metabolic actions of the receptor on fatty acid oxidation, whereas only the actions on fatty acid oxidation extend to humans. To overcome this dual role of PPARα in mice, we analyzed the action of DEHP on HFD in a humanized mouse model of PPARα that is insensitive to hepatocellular proliferation (Yang et al. 2008). In this model, the entire human *PPAR*α gene and its regulatory regions were introduced into the mPPARα null background, thereby allowing the expression, regulation, and function of human PPARα in the mouse. Although DEHP limited weight gain in WT mice as shown above, PPARα-humanized mice were not protected against diet-induced obesity (Figure 6A), demonstrating that this beneficial effect is limited to mPPARα activation. Indeed, and as expected, humanized PPARα mice did not develop the DEHP-induced hepatomegaly caused by cell proliferation (Figure 6B), but presented a slight excess of adiposity upon DEHP exposure. This was evidenced by an increase in total body mass and in epiWAT (Figure 6A,B). We thus evaluated gene expression in the liver and WAT to understand how DEHP may promote fat accumulation in humanized PPARα mice. Strikingly, although this PPARα-humanized mice model can respond to a classic PPARα agonist (Yang et al. 2008), we observed no activation of the genes and enzymatic activities controlling hepatic β-oxidation in response to DEHP (Figure 6C,D). The fact that DEHP-treated PPARα-humanized mice gained more weight than did their untreated counterparts was furthermore not caused by enhanced adipogenesis and fat storage via PPARγ activation in WAT because the expression of direct adipogenic PPARγ target genes was not modified (data not shown). Altogether, these results demonstrate that the protective effects of DEHP on weight gain are dependent on the species origin of the PPARα receptor and most probably do not extend to humans, in which the metabolic consequences of DEHP exposure, if any, should rather be detrimental.

**Figure 7:**
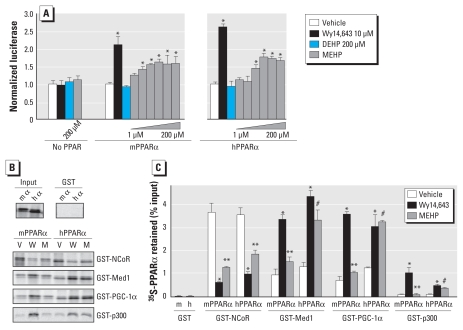
MEHP induces species-specific interactions between coregulators and mPPARα and hPPARα. (*A*) C2C12 cells grown in 12-well plates were transfected with a PPAR response element–firefly luciferase reporter construct (600 ng/well), a normalization vector encoding *Renilla* luciferase (5 ng/well), and an expression vector, either empty or coding for mPPARα or hPPARα (250 ng/well). After transfection, cells were treated with vehicle (dimethyl sulfoxide; 1‰) or the indicated ligands for 18 hr (10 μM Wy14,643, 200 μM DEHP, or increasing amounts of MEHP: 1, 3.2, 10, 32, 100, or 200 μM MEHP). Firefly luciferase activity of four biological replicates was normalized to the corresponding *Renilla* luciferase activity. (*B*) The recruitment of mouse and human ^35^S-PPARα to GST-labeled corepressor or coactivators immobilized on sepharose beads was analyzed by GST pull-down in the presence of vehicle (V), Wy14643 (W), or MEHP (M), and the material retained was quantified by SDS-PAGE followed by phosphoimager quantification. (*C*) Values are plotted as percentages of inputs, with bars indicating the average of two independent experiments. *p ≤ 0.05 compared with vehicle control. **p ≤ 0.05 compared with Wy14,643. ^#^*p*≤ 0.05 comparing the fold change of interaction between vehicle and MEHP for mPPARα with that of hPPARα.

According to the humanized mouse model used in this study, the mechanism involved in the species-specific response to DEHP seems to rely solely on differences in the receptor itself. To understand the molecular basis of this differential action, we first evaluated the ability of mPPARα and hPPARα to activate the PPAR response element of a reporter construct in the presence of the full PPAR agonist Wy14643 or of MEHP, the active metabolite of DEHP (Figure 7A). In this assay, MEHP treatment resulted in a maximal activation of mPPARα and hPPARα to a similar extent, despite having a slightly higher affinity for the mouse than for the human receptor, as previously observed (Bility et al. 2004; Hurst and Waxman 2003). To further analyze what could be the functional consequence of the subtle conformational differences of mPPARα versus hPPARα, we tested their ability to interact with coregulators in the absence of ligand or upon binding of Wy14643 or MEHP (Figure 7B). In the presence of Wy14643, mPPARα and hPPARα shared a similar pattern of interaction with coregulators, releasing NCoR (nuclear receptor co-repressor 1) and efficiently recruiting Med1 (mediator complex subunit 1), PGC-1α (peroxisome proliferator-activated receptor-γ coactivator 1α), and to a lower extent p300 (E1A binding protein p300). In contrast, MEHP induced a partial release of NCoR with both the mouse and the human receptor but failed to induce the ligand-dependent recruitment of the coactivators Med1, p300, and PGC-1α to mPPARα. In contrast, all three coactivators were efficiently recruited by the human receptor in the presence of MEHP, at a level very close to that obtained with Wy14643. Together with the experiments in the humanized PPARα mouse model, these *in vitro* observations suggest that subtle differences in the mPPARα and hPPARα receptors contribute to a species-specific PPARα-dependent metabolic response to DEHP.

## Discussion

### DEHP protects from diet-induced obesity in WT mice via hepatic PPARα activation

DEHP is the most widely used industrial plasticizer, and human exposure to this pollutant is high through the daily use of polyvinyl chloride products (NTP-CERHR 2000). Mice fed either CD or HFD supplemented with DEHP were partially protected from diet-induced obesity because they gained 30% less weight than did their controls without modifying their feeding behavior. This reduced weight gain resulted solely from reduced fat mass and was associated with a metabolic improvement, which includes lower levels of triglycerides in the liver and the blood, smaller adipocytes, and enhanced glucose tolerance. Using a combination of metabolic phenotyping, metabolomics, gene expression profiling, and genetically engineered mouse models, we could attribute this phenotype primarily to a PPARα-dependent activation of fatty acid catabolism in the liver. DEHP induced the expression of genes controlling β-oxidation of fatty acids in the liver as well as that of FGF-21, a novel PPARα target important for lipid oxidation and ketogenesis (Badman et al. 2007; Inagaki et al. 2007). We demonstrated that PPARα was required for the protective effect of DEHP by showing that both the protection from diet-induced obesity and the induction of oxidative genes in the liver were completely abolished in PPARα-null mice. Although our results clearly demonstrate DEHP-mediated metabolic interference via PPARα, other metabolic pathways—such as those driven by CAR (constitutive androstane receptor) and Rev-Erbα (nuclear receptor subfamily 1, group D, member 1)—could also be involved (Eveillard et al. 2009).

Unlike what has been hypothesized from the ability of MEHP to activate PPARγ and promote adipogenesis in cellular models (Bility et al. 2004; Feige et al. 2007; Grun and Blumberg 2007; Heindel 2003; Hurst and Waxman 2003), we found that DEHP exposure does not cause PPARγ activation and fat accumulation in the adipose tissue of WT mice. The reasons for this observation remain unclear because MEHP has been detected as the major metabolite in the blood of rats treated with doses of DEHP in the same range as those used in the present study (Akingbemi et al. 2004). In those animals, circulating MEHP concentrations were around 10 μM (Akingbemi et al. 2004), a level at which PPARγ can be activated by MEHP in cellular assays (Feige et al. 2007). Moreover, in our previous study (Feige et al. 2007), the levels of MEHP in the blood were sufficient to induce the hepatic activation of PPARα, an activation that occurs at similar concentrations for PPARα and PPARγ. Thus, the most likely hypothesis is that MEHP cannot enter adipocytes in sufficient concentrations to activate PPARγ.

### DEHP exacerbates diet-induced obesity in a humanized PPARα mouse model

It is well established that DEHP is a potent inducer of hepatocellular and peroxisome proliferation in rodents through PPARα activation by MEHP (Rusyn et al. 2006) and that these proliferative processes do not occur in humans after PPARα activation (Holden and Tugwood 1999). To overcome this limitation and to evaluate the effects of DEHP in a model more relevant to humans, we administered DEHP to mice humanized for PPARα (i.e., expressing the human PPARα receptor in the mPPARα null background) that do not exhibit hepatocellular proliferation but whose PPARα-dependent oxidative functions remain intact (Cheung et al. 2004; Yang et al. 2008). Strikingly, the metabolic actions of DEHP were abolished in this mouse model, which actually tended to be more sensitive to diet-induced obesity than were untreated controls. This unexpected phenotype may relate to two main cellular defects. First, it is possible that hepatocellular proliferation and the resultant hepatomegaly itself are important metabolic contributors. Second, and more specifically, genes responsible for fatty acid oxidation are not up-regulated in the liver of DEHP-treated humanized PPARα mice, whereas they are markedly induced in response to synthetic PPARα agonists.

Although MEHP induced a similar transactivation of mPPARα and hPPARα in cellular models, it actually induced stronger interactions with coactivators with the human receptor than with the mouse receptor in GST pull-downs. It is therefore surprising that the induction of PPARα target genes by DEHP exposure is blunted in PPARα-humanized mice. However, interactions with coregulators depend highly on the promoter context (Guan et al. 2005), and the regulation of genes crucial to metabolic homeostasis could potentially involve promoter-specific interactions with coregulators. Because activation of PPARα was recently reported to repress Let7C microRNA in WT but not in humanized PPARα mice (Shah et al. 2007; Yang et al. 2008), the blunted response to DEHP in PPARα-humanized mice may also reflect a dependency on this microRNA-mediated response. However, these hypotheses do not exclude the possibility that secondary MEHP metabolites could also play important roles in the metabolic actions of DEHP by differentially activating hPPARα and mPPARα.

### Considerations regarding the metabolic consequences of DEHP exposure in humans

The extrapolation of the action of DEHP from animal models to humans is definitely a complex issue that depends both on the relative levels of exposure and on the conservation of the underlying molecular mechanisms of action between species. Our studies involved relatively high levels of DEHP exposure, which were chosen according to previous reports of DEHP administration in rodents (David et al. 1999; Lapinskas et al. 2005). These doses are 2 to 3 orders of magnitude greater than estimated human exposures when normalized to body mass (NTP-CERHR 2000). However, the extrapolation of biological observations from rodents to humans is not linear to body mass, and levels of mouse exposure similar to those used herein lead to plasmatic MEHP concentrations in the low micromolar range (Akingbemi et al. 2004). Moreover, individuals requiring frequent blood transfusion or dialysis are subjected to repetitive acute exposures to high levels of DEHP because of the leaching of the compound from plastic bags and tubing in direct contact with biological fluids. Under such circumstances, the plasma levels of MEHP can also reach the micromolar range in humans (NTP-CERHR 2000), suggesting that the metabolic consequences of DEHP exposure described here may extend to humans. Thus, the most relevant observation is that DEHP slightly promotes weight gain in PPARα-humanized mice. Consistent with this observation, the urinary concentrations of phthalate metabolites have been recently positively correlated with obesity and insulin resistance in humans (Hatch et al. 2008; Stahlhut et al. 2007). Our results clearly demonstrate that these effects are most likely not linked to activation of PPARγ and adipogenesis in the adipose tissue but potentially to low hepatic oxidative metabolism.

## Conclusion

Altogether, our results demonstrate that exposure to the environmental pollutant DEHP has far-reaching metabolic consequences that rely on hepatic oxidative metabolism via PPARα activation. Furthermore, our study also highlights a species-specific relationship between exposure to DEHP and diet-induced obesity.
